# Bi-allelic variations in *CRB2*, encoding the crumbs cell polarity complex component 2, lead to non-communicating hydrocephalus due to atresia of the aqueduct of sylvius and central canal of the medulla

**DOI:** 10.1186/s40478-023-01519-8

**Published:** 2023-02-20

**Authors:** Aude Tessier, Nathalie Roux, Lucile Boutaud, Elodie Lunel, Leila Hakkakian, Mélanie Parisot, Meriem Garfa-Traoré, Amale Ichkou, Nadia Elkhartoufi, Christine Bole, Patrick Nitschke, Jeanne Amiel, Jelena Martinovic, Férechté Encha-Razavi, Tania Attié-Bitach, Sophie Thomas

**Affiliations:** 1 Service de Médecine Génomique des Maladies Rares, Hôpital Universitaire Necker-Enfants Malades, Paris, France; 2 INSERM UMR 1163, Institut Imagine, Université Paris Cité, Paris, France; 3 Genomics Core Facility, Institut Imagine-Structure Fédérative de Recherche Necker, INSERM U1163 et INSERM US24/CNRS UAR3633, Paris Descartes Sorbonne Paris Cite University, Paris, France; 4 Cell Imaging Platform, INSERM-US24-CNRS UMS 3633 Structure Fédérative de Recherche Necker, Paris University, 75015 Paris, France; 5 Bioinformatics Platform, Institut Imagine, Paris, France; 6 Unité de Foetopathologie, AP-HP, Hôpital Antoine Béclère, Groupe Hospitalo-Universitaire Paris Saclay, Clamart, France

**Keywords:** Congenital hydrocephalus, Ventriculomegaly, Aqueduct of sylvius atresia, Central canal of the medulla, CRB2, Cell–cell junction, Cell polarity, Apical constriction, CCDC88C, MPDZ

## Abstract

Congenital hydrocephalus is a common condition caused by the accumulation of cerebrospinal fluid in the ventricular system. Four major genes are currently known to be causally involved in hydrocephalus, either isolated or as a common clinical feature: *L1CAM*, *AP1S2*, *MPDZ* and *CCDC88C.* Here, we report 3 cases from 2 families with congenital hydrocephalus due to bi-allelic variations in *CRB2,* a gene previously reported to cause nephrotic syndrome, variably associated with hydrocephalus. While 2 cases presented with renal cysts, one case presented with isolated hydrocephalus. Neurohistopathological analysis allowed us to demonstrate that, contrary to what was previously proposed, the pathological mechanisms underlying hydrocephalus secondary to *CRB2* variations are not due to stenosis but to atresia of both Sylvius Aqueduct and central medullar canal. While CRB2 has been largely shown crucial for apico-basal polarity, immunolabelling experiments in our fetal cases showed normal localization and level of PAR complex components (PKCι and PKCζ) as well as of tight (ZO-1) and adherens (β-catenin and N-Cadherin) junction molecules indicating a priori normal apicobasal polarity and cell–cell adhesion of the ventricular epithelium suggesting another pathological mechanism. Interestingly, atresia but not stenosis of Sylvius aqueduct was also described in cases with variations in *MPDZ* and *CCDC88C* encoding proteins previously linked functionally to the Crumbs (CRB) polarity complex, and all 3 being more recently involved in apical constriction, a process crucial for the formation of the central medullar canal. Overall, our findings argue for a common mechanism of *CRB2*, *MPDZ* and *CCDC88C* variations that might lead to abnormal apical constriction of the ventricular cells of the neural tube that will form the ependymal cells lining the definitive central canal of the medulla. Our study thus highlights that hydrocephalus related to *CRB2*, *MPDZ* and *CCDC88C* constitutes a separate pathogenic group of congenital non-communicating hydrocephalus with atresia of both Sylvius aqueduct and central canal of the medulla.

**Supplementary Information:**

The online version contains supplementary material available at 10.1186/s40478-023-01519-8.

## Introduction

Hydrocephalus is defined as “an active distension of the ventricular system resulting from inadequate passage of cerebrospinal fluid (CSF) from its point of production within the cerebral ventricles to its point of absorption into the systemic circulation” [1]. Many classifications have been proposed: acquired vs. developmental, obstructive vs. communicating, syndromic vs. non-syndromic; but none of them covers all of the etio-pathogenic aspects [2]. Congenital hydrocephalus usually has a poor outcome due to irreversible cell damage during pregnancy [3]. CSF accumulation in the ventricular system may be due to CSF overproduction, inefficient reabsorption into the systemic circulation, abnormal cilium-dependent flow or complete or partial obstruction of the ventricular system. In particular, stenosis of the Aqueduct of Sylvius, that connects the third and fourth ventricles, accounts for the majority of cases of non-syndromic (no extra-cerebral feature) congenital hydrocephalus, of whom 5–15% have an X-linked form due to variations in *L1CAM* that are associated to a wide phenotypic spectrum [2]. Pathogenic variations in *AP1S2* have then been shown causal in congenital hydrocephalus, including cases with *L1CAM*-like phenotypic spectrum, which was therefore recognized as a separate X-linked syndrome, named Fried-Pettigrew syndrome (OMIM #304,340), characterized primarily by intellectual disability, basal ganglia iron or calcium deposition and hydrocephalus also associated to aqueductal stenosis. In addition, causal variations in *MPDZ* and *CCDC88C* have been associated with recessive forms of congenital hydrocephalus (OMIM #615,219 and #236,600 respectively) sharing many neuropathological similarities including atresia of both Sylvius aqueduct and central canal of the medulla, in accordance with the close functional link of both proteins. Indeed*,* CCDC88C (aka DAPLE) directly interacts with MPDZ (aka MUPP1), and they colocalize at the apical cell junction in the neural plate where they cooperate to promote apical cell constriction during neurulation [4, 5].

MPDZ also binds to PALS1, a component of the Crumbs (CRB) polarity complex which also comprises PATJ and the CRB proteins. The CRB proteins are the only transmembrane members of the CRB complex, with a short intracellular tail that establishes a scaffolding complex with other members of the CRB complex and the other two evolutionarily conserved polarity complexes: Par (another apical complex) and Scribble (a basal complex), all essential for the correct establishment of apico-basal polarity. In mammals, three CRB proteins (CRB1, CRB2 and CRB3) encoded by three distinct genes have been described. While CRB3 has a very short extra cellular domain, both CRB1 and CRB2 have a large extracellular domain composed of Laminin- and EGF-like domains [6–8].

In human, while there is no known disease associated with *CRB3*, both *CRB1* and *CRB2* bi-allelic pathogenic variations have been reported. *CRB1* has been associated with retinal dystrophy including Leber’s congenital amaurosis and retinitis pigmentosa [9], whereas *CRB2* variations are responsible for a wide phenotypic spectrum ranging from a severe prenatal disease with severe renal anomalies variably associated with hydrocephalus to postnatal isolated renal anomalies, with only few cases with retinal involvement [10–18]. To date, 34 cases with *CRB2* bi-allelic variations have been reported, including many cases with hydrocephalus, but, partly because this gene was first considered as causal for renal abnormalities, no neuropathological characterization has been performed until now. Here, we report the pathological phenotype, as revealed by fetal autopsy including neurohistopathological analysis, of 3 fetal cases with congenital hydrocephalus and harboring compound heterozygous variations in *CRB2*. In addition to confirming *CRB2* as a causal gene for hydrocephalus, our findings constitute essential cues for a pathophysiological mechanism underlying *CRB2*-driven hydrocephalus, which would be common to *MPDZ* and *CCDC88C* variations and would involve abnormalities of the apical constriction process that would ultimately lead to atresia of the central canal of the medulla and the Sylvius aqueduct.

## Methods and patients

### Methods

#### Autopsy

According to the French law, complete fetal autopsy was performed after parental consent and according to standardized protocols. Detailed examination included X-ray, photographs, macroscopic and histological examination of all viscera. Fetal biometric data were assessed according to the morphometric criteria of Guihard-Costa and colleagues [19]. All tissue specimens were immediately fixed in 10% formalin solution and processed routinely for embedding in paraffin wax. They were stained with hematoxylin, eosin and safran (HES). Then the slides were examined by light microscopy. Kidney sample were stained with Periodic Acid Schiff, and immune labeling was performed with anti-CD10 and anti-EMA. Fetal tissue samples were frozen at − 80 °C for molecular genetic tests with parental consent. Placenta was provided and examined after formalin fixation. For histological examination, blocks containing cord, membrane and full thickness of villous tissue were taken. Tissue sections of 3 μm thickness were performed on paraffin embedded biopsies and stained with HES.

#### Neuropathological examination

Brain was fixed in a 10% buffered formalin-zinc buffer solution. Weights, measurements, external gross appearance (meninges, gyration, major structures of the skull base, aqueduct of Sylvius, brainstem, cerebellum) were documented. The cerebral hemispheres were sectioned in the coronal plane to assess the extent of ventricular dilatation and relevant anatomy. The midbrain and hindbrain samples were sectioned transversally to ensure adequate inspection of the Sylvius aqueduct. For histological analysis, 7-μm sections were cut and stained with hematoxylin and eosin (H&E).

#### Immunohistochemistry

Double immunolabelings were performed on 7 µm thick sections of midbrain and hindbrain from fetus 3 and an age-matched control using ZO-1 (Thermofisher 617,300), PKCι (BD Biosciences 610,175), PKCζ (Santa Cruz SC17781), β-catenin (BD Biosciences 610,154) and N-cadherin (BD Biosciences 610,921) antibodies (Additional File 1: Table S1). Confocal images were taken on a Zeiss LSM 700 microscope and were analyzed with ImageJ software.

#### Exome sequencing

DNA was extracted from muscle or lung using a manual salting-out method for the fetuses and from blood samples for the parents. Exome capture was performed at the genomic platform of the IMAGINE Institute (Paris, France) using the SureSelect Human All Exon kit CRE (Clinical Research Exome) or SureSelect Human All Exon kit 58 Mb V6, Agilent Technologies). Exome libraries were prepared from 3 µg of genomic DNA sheared with an Ultrasonicator (Covaris, Woburn, MA) as recommended by the manufacturer. Barcoded exome libraries were pooled and sequenced using a HiSeq2500, Illumina generating paired-end reads. After demultiplexing, sequences were mapped on the human genome reference (NCBI build37/hg19 version) with BWA. The mean depth of coverage obtained for each sample was >  = 120X with > 97% of the exome covered at least at 15X and > 94.7% covered at least at 30X. Variant calling was carried out with the Genome Analysis Toolkit (GATK), SAMtools, and Picard Tools. Single nucleotide variants were called with GATK Unified Genotyper, whereas indel calls were made with the GATK IndelGenotyper_v2. All variants with a read coverage ≤ 2 × and a Phred-scaled quality of ≤ 20 were filtered out. All the variants were annotated and filtered using an in-house developed annotation software system. We first focused our analyses on non-synonymous variants, splice site variants, and coding indels. Variants’ pathogenicity was evaluated using SIFT (cutoff ≤ 0.05), PolyPhen2 (HumVar scores, cutoff ≥ 0.447) and Mutation Taster (cutoff: qualitative prediction as pathogenic) prediction algorithms. We also assessed frequency in control populations and datasets including the ExAC database, dbSNP129, the 1000 Genomes Project, ClinVar, HGMD and in-house exome data. Evolutionary conservation scores were obtained through PhyloP and GERP++ . Sanger sequencing was performed on Fetus 3 parent’s DNA to analyze the segregation of the variations (primers are available upon request).

### Cases

#### Family 1

**Fig. 1:**
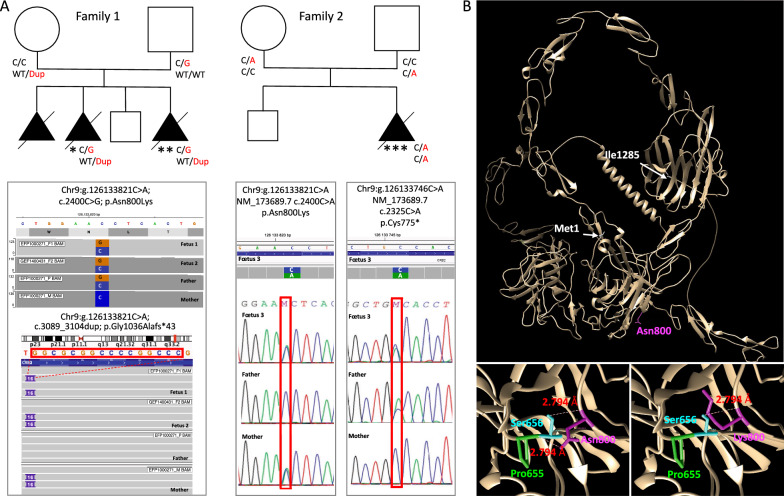
*CRB2* variations in the 2 reported families. **A** Pedigree of both families with 3 female fetuses carrying bi-allelic compound heterozygous *CRB2* variations. *CRB2* variants are reported on the basis of Human Genome Assembly GRCh37 (hg19), and the *CRB2* reference sequence used was GenBank: NM_173689.7. Fetus 1 (*) and 2 (**) from family 1 harbor two compound heterozygous variations c.2400C > G p.(Asn800Lys), inherited from their father and c.3089_3104dup p.(Gly1036Alafs*43) inherited from their mother. Fetus 3 (***) from family 2 harbor two compound heterozygous variations c.2325C > A p.(Cys775*) inherited from the father and c.2400C > A p.(Asn800Lys) inherited from the mother. **B** 3D modeling of human CRB2 (Uniprot Q5IJ48) based on the AphaFold structure prediction and using UCSF Chimera showing that Asn800 is involved in two hydrogen binding interactions with Ser656 and Pro655 and that modifying Asn800 to Lys leads to the loss of the hydrogen bond with Pro655 suggesting altered stability of the mutated protein

An unrelated caucasian couple had a first pregnancy terminated after the discovery of hydrocephalus on ultrasound (US) examination, fetal examination wasn’t performed. Based on the recurrence of hydrocephalus and in accordance with French legislation, the second pregnancy was also terminated at 19 weeks of gestation (WG) (fetus 1). Four years later, a third medical termination of pregnancy was performed at 18 WG after the discovery on US examination of biventricular dilatation, bilateral hyperechogenic kidney and midline anomalies (fetus 2). The level of α-fetoprotein in maternal blood or amniotic fluid was not measured. The familial pedigree is depicted in Fig. 1.

#### Family 2

The third case (fetus 3) is a 25 WG female fetus from unrelated caucasian parents. First trimester screening showed elevated α-fetoprotein but a low risk for trisomy 21, with normal cranio-caudal length (82 mm) and nuchal translucency (1.3 mm) at US examination. However, a second US examination at 23 WG revealed dilatation of lateral ventricles with ventricular septal rupture and macrocephaly. No additional anomaly was observed. Fetal brain MRI confirmed these findings and further revealed mild dilatation of the third ventricle and thinning of the otherwise complete corpus callosum. There was no evidence of hemorrhage. Array CGH on amniotic fluid was normal. Based on these findings, a medical termination of pregnancy was performed at 25 WG. The familial pedigree is depicted in Fig. 1.

## Results

### Fetus 1

**Fig. 2:**
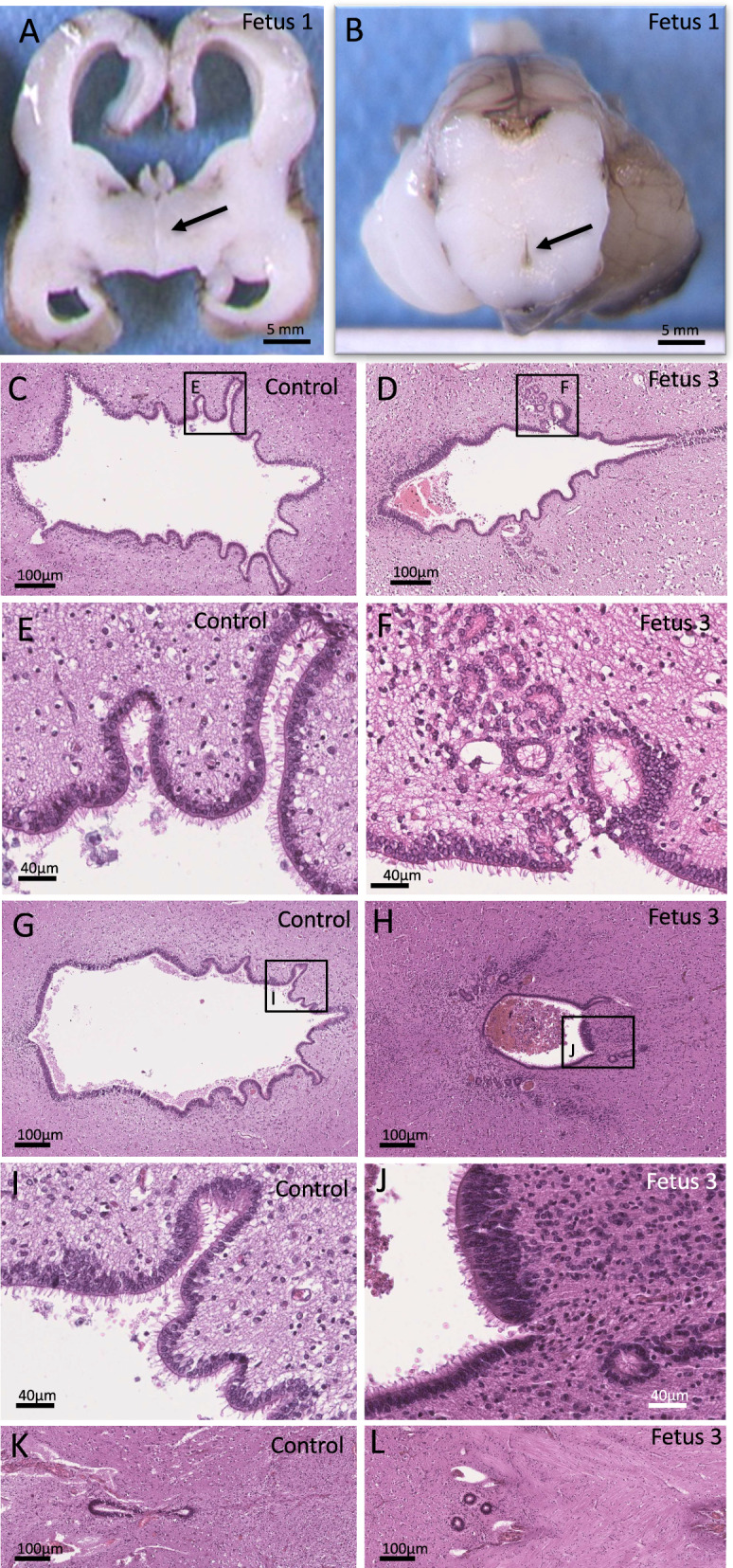
Main neuropathological findings in fetuses with *CRB2* pathogenic variants. Macroscopic coronal section of the brain of fetus 1 showing severe bilateral ventricular dilatation, thinning of the cerebral parenchyma and undiscernible third ventricle (arrow) (**A**). Macroscopic transversal section of the midbrain showing atretic aqueduct of Sylvius while the pons and cerebellum appear normal (**B**). Hematoxylin and eosin staining of transversal sections along the mesencephalon and medulla of a control fetus (**C**, **E**, **G**, **I**, **K**) and fetus 3 (**D**, **F**, **H**, **J**, **L**) showing small dysmorphic aqueduct with multiple indentations and rosettes (**D**, **H**) with otherwise normally ciliated ependymal cells in fetus 3 (**F**, **J**) as compared to the age-matched control (**C**, **E**, **G**, **I**). Transversal section of the medulla showing atresia of the central medullar canal, reduced to 3 rosettes (**L**) as compared to an age-matched control in which the central canal of the medulla is normally visualized (**K**)

Post mortem examination found a eutrophic female fetus with camptodactyly, without any macroscopic visceral malformation. No dysmorphic features were noted. X-rays performed to detect bone anomalies were unremarkable. At histology, medullar cysts of varying size and localized on distal tubules were observed in both kidneys. Neuropathological examination found biventricular dilatation with ependymal abrasion. Third ventricle, Sylvius aqueduct and fourth ventricle were atretic, with ependymal anomalies, rosettes and macrophagic reaction (Fig. 2A and B). Cortico-spinal tracts were conserved. On lower sections containing the medulla, the central canal of the medulla was reduced to a thin slot with multiple rosettes. H&E staining of the eyes reveals normal histology, in particular there were no retinal rosettes (data not shown).

### Fetus 2

Post mortem examination found a eutrophic female fetus with macrocephaly, mild retrognathia and bilateral kidney hypoplasia. No specific craniofacial dysmorphic feature was reported. X-rays did not reveal any skeletal anomalies. At histology, medullar cysts of varying size localized on distal tubules were identified in both kidneys. Neuropathological examination found severe biventricular dilatation with diffuse ependymal abrasion. The third ventricle, the aqueduct of Sylvius and the fourth ventricle were atretic, with rosette formation and macrophagic reaction. The cerebellum was otherwise normal.

Exome sequencing was performed in both affected fetuses and their parents. Two variations were identified in both fetuses. The first variation involves the exon 8 of *CRB2* ((NM_173689.7): c.2400C > G p.(Asn800Lys)) and is inherited from the father while the second variation is a 16 base pairs (bp) duplication in exon 10 ((NM_173689.7): c.3089_3104dup p.(Gly1036Alafs*43)) inherited from the mother. The missense variation was already described in the literature in patients with ventriculomegaly [11, 13]. It affects a highly conserved amino acid and is predicted deleterious for the function of the protein by Polyphen2, SIFT and CADD (v1.6, Phred: 23.4). 3D modeling of human CRB2 (Uniprot Q5IJ48) using AphaFold structure prediction (AF-Q5IJ48-F1), shows that Asn800 is involved in two hydrogen binding interactions with Ser656 (Ser656.A OG-Asn800.A O 2.794 Å) and Pro655 (Asn800.A ND2-Pro655.A O 2.783 Å). Changing Asn800 to Lys800 leads to the loss of the hydrogen bond with Pro655 potentially affecting the stability of the protein (Fig. 1b). The 16 bp duplication is also a recurrent variation, previously reported in a patient with ventriculomegaly [10]. This duplication leads to a frameshift and the creation of a premature termination codon with subsequent potential targeting of the mutated transcript for nonsense-mediated mRNA decay (NMD). While both variations are reported heterozygous in gnomAD, in 58 and 28 individuals respectively for the missense and the duplication, none of them was reported homozygous in the database.

### Fetus 3

Post mortem examination found macrocephaly and a sacral dimple. Neither specific dysmorphic features nor visceral abnormality was reported. Limbs were normal, in particular there was no adducted thumbs or camptodactyly. No abnormality was detected at histological visceral examination.

Brain weight was in accordance with the term, despite hydrocephalus. On external examination, gyration was concordant with the term and olfactory bulbs as well as optic chiasma were present. On supratentorial coronal sections, ventricular dilatation appears severe, with a considerable thinning of the cerebral parenchyma and septal rupture. The third and fourth ventricles were severely narrowed. On transversal sections of the mesencephalon, Sylvius aqueduct was undiscernible. At histology, we identified atresia-forking of the aqueduct of Sylvius, defined by obliterations of the aqueduct which is replaced by normally multiciliated ependymal cells scattered in rosette-like structures (Fig. 2D, F, H, J) as compared to an age-matched control (Fig. 2C, E, G, I). On the most proximal transversal sections, the aqueduct had an abnormal central localization with a dysmorphic and reduced aspect. Clustered or dispersed inflammatory CD68+ cells were observed around the aqueduct of Sylvius, suggesting a macrophagic reaction (Additional File 1: Fig. S1). The fourth ventricle wall also presented ependymal lesions with rosettes. More distally, the central canal of the medulla was only composed of rosette-like structures (Fig. 2L). On the supra tentorial part, the cortical lamination, the internal capsule and the cortico-spinal tract were normal. Surrounding the ventricular layer, some subependymal heterotopias are observed. They are made of small granular cells with dark round nuclei compatible with progenitor cells, evocative of periventricular immature cells. Indeed, immunolabeling experiments with GFAP antibody showed that most of these cells are GFAP-, while larger cells reminiscent of astroglial cells are GFAP+ (Additional File 1: Fig. S2). Multiple loci of ependymal abrasions were also seen. Both lesions may be secondary to the high intracranial pressure. H&E staining of the eyes reveal no disorganization of the neural retina. Whole exome sequencing identified two compound heterozygous variations in the exon 8 of *CRB2* (NM_173689.6): c.2325C > A; p.(Cys775*) and c.2400C > A; p.(Asn800Lys). The stop variation was never reported in any database, neither in patients nor in healthy individuals and is expected to lead to the degradation of the mutated transcript by NMD. The missense variation involves the same nucleotide as in family 1, that is substituted to a different nucleotide (here C > A versus C > G in family 1) but leading to the same amino acid modification (Asn to Lys), and thus with the same predicted consequences on the protein stability (Fig. 1B). It was reported heterozygous in 4 individuals in gnomAD but never homozygous. Variations and their segregation were confirmed by Sanger sequencing indicating that the stop variant was inherited from the father while the missense variation was inherited from the mother.

**Fig. 3:**
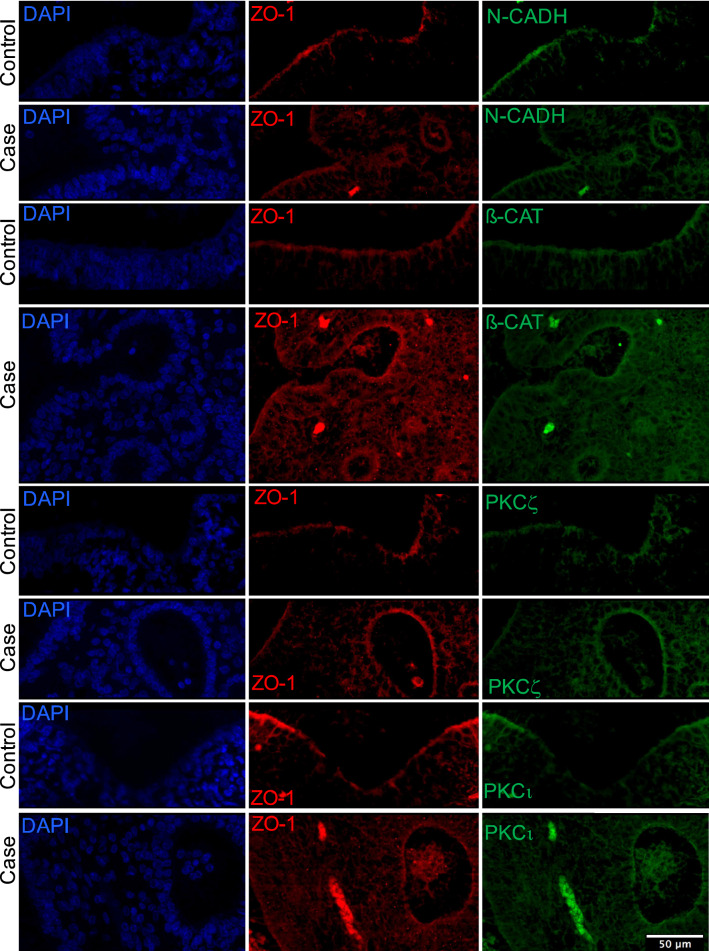
Confocal analysis performed after double immunostaining on sections of the midbrain from fetus 3 and an age-matched control with ZO-1 antibody (red) combined with either N-cadherin (green) or β-catenin (green) or PKCζ (green) or PKCι (green) showing that mutated and control fetuses show similar staining for all markers

Double immunolabeling analysis on transverse sections of the mesencephalon revealed normal localization of the tight junction protein (ZO-1), the adherens junction proteins (β-catenin and N-Cadherin) and the atypical protein kinases C (PKCι, PKCζ) of the PAR polarity complex on the ventricular cells. In addition, no significant differential expression between control and patient was observed (Fig. 3). Together, these findings suggest normal apico-basal polarity and cell–cell adhesion of the neuro-epithelium.

## Discussion

*CRB2* variations were first identified in patients with isolated steroid resistant nephrotic syndrome [10] and in patients with congenital nephrosis with cerebral ventriculomegaly [11, 13]. *CRB2* was indeed originally largely associated to renal anomalies including renal tubular or glomerular microcysts detected at microscopic examination. Interestingly, one of our case did not show any renal features as already reported in other cases [11], making hydrocephalus one of the major signs associated to bi-allelic *CRB2* variations and highlighting *CRB2* as one of the major causative gene for hydrocephalus. Among the previously described cases, 5 were reported with Sylvius Aqueduct stenosis as the cause of hydrocephalus, but none of these cases had a neuropathological examination, only MRI. While imaging analysis such as ultrasound scans or MRI are very useful tools to evaluate brain malformations during pregnancy, only neuropathology allows to differentiate Sylvius Aqueduct stenosis from atresia, which are two different malformations with distinct etiologies. Our work thus clarifies the etiology of the cerebral phenotype associated to *CRB2* variants, revealing that unlike Sylvius Aqueduct stenosis where the third ventricle is usually enlarged, in *CRB2* mutated cases, only the lateral ventricles are dilated, the downstream tract being atretic from the third ventricle to the medulla. This neuropathological feature was highly suggestive of *MPDZ* variations [20] which have been associated with non-syndromic hydrocephalus due to Sylvius Aqueduct atresia, distinguishing them from cases with *L1CAM* variations, in which hydrocephalus was associated with Sylvius Aqueduct stenosis. More recently, the same team reported the neuropathological phenotype of fetuses with *CCDC88C* pathogenic variants. Both described fetuses had atresia of both the Sylvius Aqueduct and the canal of the medulla with additional hydrops of the choroid plexuses. Mesencephalic and medullar lesions were also very similar to our cases [21]. Thus, importantly, such specific features, in particular the absence of third ventricle dilatation, may be seen during prenatal US scan and could be used to guide prenatal genetic testing. In accordance with such similar neuropathological characteristics, MPDZ, CCDC88C and CRB2 are functionally linked, and are crucial for the establishment of apicobasal polarity as well as cell–cell junction formation and maintenance. In this view, our immunohistochemical analysis revealed normal localization and level of proteins of the PAR polarity complex as well as components of the tight and adherens junctions suggesting normal apicobasal polarity and adhesion of the epithelial cells.

In view of the proposed function of CRB2 in preventing neocortical anomalies, we next analyzed neocortical lamination and found no defect in our fetal cases as compared to controls. However, immunohistochemistry analysis using specific antibodies allowing to discriminate specific cortical layers would ensure the absence of cortical lamination defect. In addition, specific ablation of *Crb2* in the mouse telencephalon leads to cortical lamination abnormalities that are transient [22], indicating that early transient anomalies cannot be excluded in patients with *CRB2* variations that might partially underlie intellectual deficiency and seizures.

**Fig. 4:**
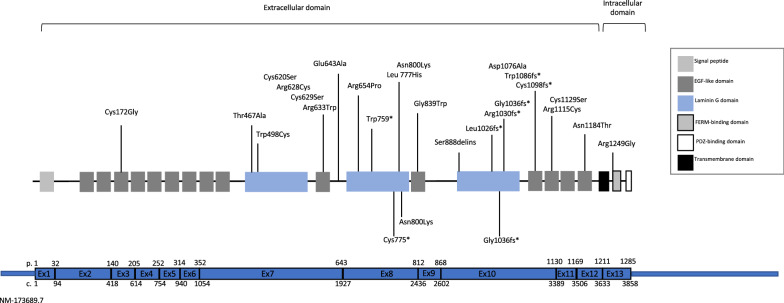
Schematic representation of CRB2 protein showing its known functional domains and the position of all variations previously reported in the literature (above) and the three variations we report in this study (below)

Concerning the eye, various functional analyses argue for an important role of CRB2 in the development and maintenance of the neural retina as revealed by the appearance of retinal rosette-like structures in *Crb2-/-* mice [23, 24]. We thus underwent a histopathological analysis of the eyes in all our cases that revealed no histological anomaly, especially no folds or rosettes of the neural retina. Besides the possibility of redundant functions of human CRB proteins in the retina, we cannot exclude late onset retinal involvement, implying the need for ophthalmological follow-up of *CRB2* mutated patients. In the literature, only a few patients with bi-allelic *CRB2* variations have been reported with retinitis pigmentosa, and they all share the same homozygous missense variation (p.Arg1249), that was shown in vitro to accelerate epithelial mesenchymal transition with subsequent degeneration of retinal pigment epithelium cells [15]. As this is the only variation reported so far in the intracellular region of the protein, a specific effect may be suspected. Interestingly, when compound with a variation affecting the extracellular domain, this variation leads to tubulopathy without retinitis pigmentosa (Fig. 4) [10]. Recently, a phenotype-genotype correlation has been suggested in view of the clustering of pathogenic variations in exons 8 and 10 in patients presenting with kidney anomalies associated to hydrocephalus, whereas variations in exons 12 and 13 seems to be associated with isolated renal disease [17]. Our data are consistent with this hypothesis, indeed our patients also had variants in exons 8 and 10, but one of them had isolated hydrocephalus.

In view of the association of renal anomalies, hydrocephalus/ventriculomegaly and retinal dystrophy, *CRB2* has been proposed to lead to a phenotypic spectrum characteristic of a ciliopathy [13]. Ciliopathies refer to a wide range of genetic diseases due to altered function of proteins that localize to motile and/or primary (immotile) cilia or to centrosomes. Because of the specific properties of primary versus motile cilia which are respectively unique, largely ubiquitous and associated with signal transduction, versus multiple, present on highly specialized cells to move fluids or to propel cells, the physiological consequences of defects in motile and primary cilia are different [25, 26]. However, both motile and primary cilia have been involved in hydrocephalus, with different underlying mechanisms [27–29]. Here, we observed a priori normal motile cilia harboring the apical side of the ependymal cells lining the central canal of the medulla arguing for a distinct mechanism affecting the formation of the Sylvius aqueduct and central canal of the medulla, although potential anomalies of the primary cilium cannot be excluded [30]. Formation of this canal is going through a complex process with remodeling of the pseudostratified ventricular layer cells which undergo apical constriction, a cell shape change involving CRB2 as well as CCDC88C and MPDZ that have been shown to work cooperatively to drive apical constriction of the neural plate cells during neurulation [4, 5]. CRB2, through its intracellular FERM-binding domain, interacts with actin-binding proteins including moesin [31, 32] and is indeed connected to the actin-myosin network essential for the conversion of the primitive lumen into the central canal of the medulla. Excitingly, CRB2 has recently been proposed to act through interaction with a locally secreted CRB2 variant modifying cell polarity and cohesion of the ventricular layer to allow its remodeling [33]. Thus, CRB2 loss might lead to altered regulation of apical constriction of the ventricular layer that might lead to invagination and rosette formation instead of delamination normally enabling dorsal collapse. Together, these studies and our findings argue for a common pathological mechanism underlying hydrocephalus associated to variations in all 3 genes that indeed lead to very similar neuropathological outcomes.

To note, elevated levels of α-fetoprotein in maternal serum and in amniotic fluid were frequently reported during pregnancy of cases with *CRB2* variations as observed here for the fetus 3. This data was not available for the other cases. While unexplained, this biological anomaly might be useful to guide the molecular diagnostic.

Overall, our work highlights the major benefit of performing neuropathological examination in fetuses and accurately describing the phenotype of rare diseases to improve the understanding of the underlying pathological mechanisms. Hydrocephalus associated to bi-allelic variations in *CRB2*, *MPDZ* and *CCDC88C* constitutes a separate pathogenic group of congenital non-communicating hydrocephalus with Sylvius aqueduct and central canal of the medulla atresia that might be linked to disturbed apical constriction of the ventricular layer cells, a complex remodeling process required for the formation of the central canal of the medulla.

### Web resources

https://alphafold.ebi.ac.uk

https://www.cgl.ucsf.edu/chimera/

CADD (v1.6): https://cadd.gs.washington.edu

gnomAD: https://gnomad.broadinstitute.org

OMIM: https://omim.org

Polyphen2 (v.2.2.2): http://genetics.bwh.harvard.edu/pph2/

SIFT (v6.2.0): https://sift.bii.a-star.edu.sg/www/SIFT_aligned_seqs_submit.html

UniProtKB: https://www.uniprot.org

https://www.nextprot.org/entry/nx_q5ij48/structures

## Supplementary Information

**Additional file 1**:**Fig. S1** CD68 and GFAP immunostainings. **Fig. S2** Periventricular heterotopias.**Table S1** Antibodies used for confocal analysis.

## Data Availability

The datasets generated and analyzed during the current study are not publicly available due to individual privacy, but are available from the authors upon reasonable request.
