# Supplementary material for: Bi-allelic variations in CRB2, encoding the crumbs cell polarity complex component 2, lead to non-communicating hydrocephalus due to atresia of the aqueduct of sylvius and central canal of the medulla
Source: Acta Neuropathol Commun. 2023 Feb 20;11:29. doi: 10.1186/s40478-023-01519-8 (PMC9940441; doi:10.1186/s40478-023-01519-8)
Supplement: Supplementary file 1 — Additional file 1:Fig. S1 CD68 and GFAP immunostainings. Fig. S2 Periventricular heterotopias.Table S1 Antibodies used for confocal analysis. [file 40478_2023_1519_MOESM1_ESM.pdf]

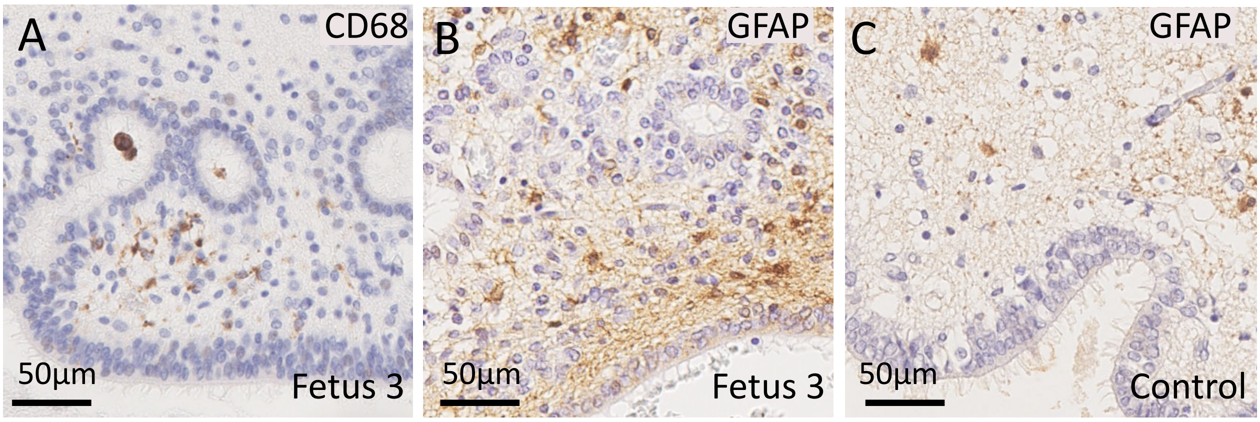

Supplementary figure 1: (A) Some CD68+ cells surround the Aqueduct of Sylvius indicative of an inflammatory reaction. Immunolabelling experiments did not show any difference in GFAP signal in cells along the ependymal lining and within rosettes in foetus 3 (B) as compared to the GFAP signal detected in ependymal cells in an age-matched control fetus (C). Of note, a substantial GFAP+ signal is detected in the subependymal region in fetus 3 in accordance with an astroglial reaction.

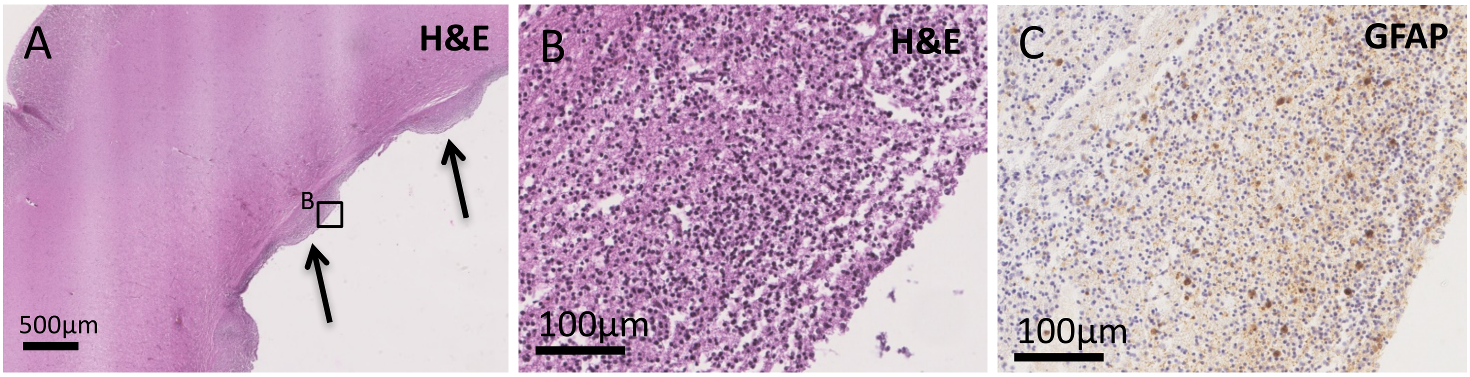

Supplementary figure 2 : (A) Periventricular nodular heterotopias are composed of granular cells with histological features of progenitors from the germinative zone that are intermingled with larger astroglial-like cells (B). Immunostaining with GFAP antibody shows a mixed population of GFAP+ astroglial cells and GFAP- immature cells reminiscent of neuronal progenitors (C). H&E: Hematoxylin and eosin, GFAP: Glial fibrillary acidic protein.

Antibodies	Clone	Dilution	Manufacturer	Targets location
ZO-1	617300	1/500	Thermofisher	tight junction
PKCι	610175	1/500	BD Bioscience	PAR polarity complex
PKCζ	SC17781	1/500	Santa Cruz	PAR polarity complex
β-catenin	610154	1/500	BD Bioscience	adherens junction
N-cadherin	610921	1/500	BD Bioscience	adherens junction

Supplementary table 1: Antibodies used for confocal analysis.
